# Color Stability and Photo-Degradation of Steamed Beech Wood with False Heartwood Under UV Exposure

**DOI:** 10.3390/polym18080984

**Published:** 2026-04-17

**Authors:** Michal Dudiak, Eva Výbohová, Ladislav Dzurenda

**Affiliations:** Faculty of Wood Sciences and Technology, Technical University in Zvolen, T.G. Masaryka 24, 960 01 Zvolen, Slovakia; vybohova@tuzvo.sk (E.V.); dzurenda@tuzvo.sk (L.D.)

**Keywords:** beech wood, false heartwood, steaming, color stability, UV radiation, ATR-FTIR spectroscopy

## Abstract

This work analyses the influence of hydrothermal treatment (steaming) on the color stability and photochemical degradation of beech wood (*Fagus sylvatica* L.) with false heartwood under the influence of UV radiation. Samples in the native state and after steaming at temperatures of 105 °C (Mode I) and 120 °C (Mode II) were exposed to simulated aging in a Xenotest device for 360 h. Color changes were assessed in the color space CIE L*a*b* and surface chemical changes using ATR-FTIR spectroscopy. The results showed that unsteamed wood darkens significantly under the influence of UV radiation (ΔL* = −10.2), while wood steamed at 120 °C shows the opposite trend—lightening (ΔL* = +8.8). The color difference ΔE* reached values of 12 to 16 units for unsteamed wood, which indicates a complete color change. Steaming at higher temperatures successfully homogenizes the color of the sapwood and false heartwood and ensures their subsequent uniform visual aging.

## 1. Introduction

Beech wood (*Fagus sylvatica* L.) is classified as a diffusely porous wood species without natural heartwood, which may develop false heartwood at an older age. It is a brown-to-red wood with a higher content of lignin and some groups of extractives, which differs from sapwood not only in color but also in texture. Although it sometimes contains less of these compounds, it still exhibits different physical and chemical properties [1]. The formation of false heartwood is influenced by ecological conditions and the growth environment of the tree, with the key factor being the penetration of air into the trunk through wounds on the bark or branches. This process leads to the oxidation of carbohydrates and starches in living or partially dead cells, which results in the formation of polyphenolic compounds. These subsequently spread to the surrounding tissue and cause its typical brown-to-red color. The differences between sapwood and false heartwood have a fundamental impact on wood processing, including steaming technology [2,3,4,5,6,7,8,9,10].

Wood steaming is a technological process in which wood is treated with saturated water steam in high-temperature closed devices, such as autoclaves. Its main aim is to improve the technological properties of wood, such as its flexibility, color uniformity, and reduction of internal stresses. When exposed to steam at temperatures from 100 to 140 °C, some cell wall components, especially lignin and hemicelluloses, are hydrolyzed, which leads to a change in the structure of the wood. Already in the initial stages of heat treatment, partial hydrolysis of hemicelluloses and the release of water-soluble substances take place. These chemical transformations depend on the temperature and duration of the process and lead to the gradual decomposition of polysaccharides. Under the influence of the acids present, such as acetic and formic acids, the oxidation of carbohydrates and pectins occurs, which dehydrates pentoses and forms 2-furaldehyde [11,12,13,14].

At the same time, changes in the lignin structure occur, where quinonemetide structures, free radicals, and phenolic hydroxyl groups are formed, which leads to the formation of new quinone-type structures. These changes are manifested by visible darkening of the wood, which affects not only its appearance, but also its physical and mechanical properties [11,12,15,16,17].

The color of wood is one of the key physical and aesthetic properties which significantly affects its use, especially in the furniture industry and in the production of floors and other decorative elements. Its character is determined by the presence of various chemical components, such as lignin, tannins, and other organic substances contained in the cellular structure of wood. The steaming process causes chemical reactions that change these components, which is subsequently manifested by a change in the color of the wood. In the case of beech wood, these changes can be significant—while the light part of the wood (sapwood) tends to fade, the false heartwood can either darken or lighten, depending on the steaming parameters, such as temperature and duration of the process [18,19].

The color of wood also changes due to long-term exposure to sunlight on its surface. Under the influence of radiation, the surface of the wood most often darkens and acquires a yellow-to-brown hue. Many scientific works deal with this change in the color of wood [20,21,22,23,24,25], or after accelerated weathering [14,16,20,26,27,28,29,30,31,32,33].

The sunlight that hits the wood is partly reflected and partly absorbed. The infrared component of the spectrum is converted into heat, while UV radiation and part of visible light (wavelength λ = 200–400 nm) initiate photochemical reactions, primarily photolytic and photooxidative processes, which take place mainly in lignin, but also in polysaccharides and other accompanying substances of wood. Lignin is the most susceptible to photodegradation, absorbing 80–85% of UV radiation, while carbohydrates absorb 5–20% and accompanying substances approximately 2% [21]. These reactions lead to the cleavage of lignin macromolecules, resulting in the formation of phenolic hydroperoxides, free radicals, and carbonyl and carboxyl groups. To a lesser extent, polysaccharides also undergo depolymerization, which breaks down into molecules with a lower degree of polymerization, while gaseous products such as CO, CO_2_, and H_2_ are also formed [15,20,22,23,24,34,35,36].

The aim of this work is to analyze the color stability of beech wood with false heartwood after steaming with saturated water vapor, through simulated aging using UV radiation in the Xenotest Q-SUN Xe-3-H device Q-Lab Corporation, Westlake, OH, USA. Changes in wood color are evaluated using the CIE L*a*b* color space coordinates, the total color difference ∆E*, and ATR-FTIR spectroscopy.

## 2. Materials and Methods

### 2.1. Material

For the purposes of the research, 25 beech trunks (length 6.2 m) exhibiting false heartwood were selected. The timber was sourced from the Central Slovakian region, specifically from the Štiavnické vrchy locality (GPS coordinates: 48.4161247 N, 18.8593111 E). Subsequently, beech blanks with false heartwood with a moisture content of w_p_ = 58.1 ± 3.7% were produced by longitudinal and transverse sawing with the following dimensions: thickness: h = 40 mm, width: w = 200 mm, length: d = 900. In total, 15 pieces for each mode were steamed in a pressure autoclave: APDZ 240 at Sundermann s.r.o. (Banská Štiavnica, Slovakia), according to the modes listed in Table 1, in order to unify the color difference between the light sapwood and the darker zone of the false heartwood. The principle of the procedure and the conditions of the technological process of steaming beech wood are given in the publication [37].

Both unsteamed and steamed beech blanks were dried to a final moisture content of w = 10 ± 0.5%, using a low-temperature regime to preserve the treatment-induced color. Drying was performed in a KAD 1 × 6 conventional hot-air dryer (KATRES s.r.o., Jihlava, Czech Republic) following a specific two-stage protocol [38]. After drying, the blanks were machined using a JET JPT-410HH milling machine (STROJE Slovensko, Banská Bystrica, Slovakia) equipped with a helical cutter head. This process achieved a high-quality surface with a mean roughness (Ra) of 3.0 to 5.0 µm, ensuring optimal conditions for subsequent optical measurements. Experimental samples with dimensions of h = 15 mm, width w = 100 mm, and length d = 100 mm were prepared, with each specimen containing both sapwood and false heartwood zones for color fastness testing.

### 2.2. Color Measurement of Beech Wood

The surface color of unsteamed and steamed beech wood samples with false heartwood was analyzed in the CIE L*a*b* color space using a tristimulus colorimeter Color Reader CR-10 (Konica Minolta, Tokyo, Japan). The color space CIE L*a*b* is three-dimensional and covers the entire range of human color perception. It is based on the opposing color model of human vision, where red and green form an opposing pair and blue and yellow form an opposing pair. This makes CIE*a*b* a Hering opposing color space. The nature of the transformations also characterizes it as a color space with a chromatic value. The lightness value, L*, defines black at 0 and white at 100. The a* axis is relative to the opposing colors of the green–red color, with negative values towards green and positive values towards red. The b* axis represents the opposing colors of the blue–yellow color, with negative numbers towards blue and positive numbers towards yellow. Measurements were performed using a D65 light source, an 8 mm diameter optical sensing aperture, and a CIE 10° standard observer, which is recommended for evaluating wood surface color due to its better correlation with visual perception on larger areas. The color measurements were performed on the radial surfaces of the wood samples before and after irradiation.

The color evaluation of beech wood with false heartwood in the CIE L*a*b* color space before irradiation is shown in Table 2.

The overall color variation ΔE* of beech wood surfaces containing false heartwood, induced by UV exposure, was calculated in accordance with the ISO 11664-4 standard [39] using the following formula:

(1) $$\Delta E^{*}=\sqrt{{\left(L_{360}^{*}-L_{0}^{*}\right)}^{2}+{\left(a_{360}^{*}-a_{0}^{*}\right)}^{2}+{\left(b_{360}^{*}-b_{0}^{*}\right)}^{2}}$$

where L*_0_; a*_0_; b*_0_ values in the color space coordinates of the surface of dried milled non-steamed and steamed beech wood before exposure;

L*_360_; a*_360_; b*_360_ values in the color space coordinates of the surface of dried milled non-steamed and steamed beech wood after exposure to UV radiation in the xenotest [39].

### 2.3. Exposure of Beech Wood to UV Radiation in a Test Chamber

To simulate indoor UV degradation, samples were exposed to a full-spectrum xenon arc lamp (1800 W) in a Q-SUN Xe-3-H test chamber for a total duration of τ = 360 h. The environmental parameters were set to a dry mode with a black panel temperature of 63 °C and an irradiance of 0.35 W/m^2^—340 nm. Colorimetric data were collected systematically throughout the irradiation period at τ = 24 h increments.

### 2.4. Determination of Chemical Changes in Wood Using ATR-FTIR Spectroscopy

The chemical changes on the surface of the wood samples induced by UV irradiation were analyzed using FTIR spectroscopy. The measurements were performed with a Nicolet iS 10 FTIR spectrometer (Thermo Fisher Scientific, Madison, WI, USA) equipped with an attenuated total reflectance (ATR-FTIR) accessory featuring a diamond crystal. The following scanning parameters were applied: a wavenumber range of 4000–650 cm^−1^, 32 scans per measurement, and a spectral resolution of 4 cm^−1^. The obtained spectra were evaluated using the OMNIC 9 spectroscopic software. The band ratios (e.g., H_1592_/H_1370_) were calculated from the peak heights (H) of the respective absorption bands.

### 2.5. Processing and Formal Statistical Testing of Experimental Data

To assess the correlation between different sample sets, the experimental data were processed using the TIBCO Statistica 14.0 program (TIBCO Software Inc., Palo Alto, CA, USA). The assessment involved both *t*-tests and ANOVA to determine if observed color changes were statistically significant. All relationships were interpreted based on the calculated *p*-values, and the resulting trends were visualized graphically.

## 3. Results and Discussion

**Figure 1:**
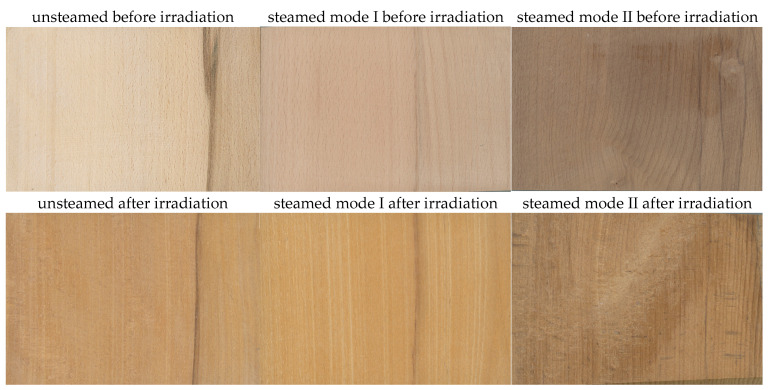
Visual comparison of the surface of beech wood with false heartwood in the native state and after steaming (105 °C and 120 °C) before and after UV irradiation in the Q-SUN Xe-3-H test chamber.

The color of unsteamed and steamed beech wood with false heartwood before and after UV irradiation in the Q-SUN Xe-3-H test chamber is shown in Figure 1.

Visual evaluation of samples exposed in the Q-SUN Xe-3-H chamber confirmed that the reaction of the wood surface to UV radiation is critically dependent on its previous heat treatment.

In unsteamed beech wood, we observed a significant loss of the original light white-yellow-gray color. Due to the influence of photochemical reactions, the surface visibly darkened and acquired a rich yellow-brown hue. This phenomenon is explained by [15,40] by the degradation of lignin and extractives, which creates new chromophoric groups, mainly of the quinone type, that intensively absorb visible light in the blue region of the spectrum, which we perceive as yellowing and browning.

The steaming process fundamentally changes not only the initial color of the wood, but also the kinetics of its changes during simulated aging: Mode I (105 °C): The original pale pink-brown color obtained by steaming showed a slight darkening and shift to brown-yellow shades after exposure. In terms of visual stability, this mode appears to be more stable than native wood, which is also confirmed by the lower values of the total color difference ΔE* compared to unsteamed sapwood. Mode II (120 °C): We noted the most interesting trend in samples steamed with saturated water steam at a temperature of 120 °C. The originally saturated brown-red color lightened under the influence of UV radiation. This phenomenon of lightening (increase in the L* coordinate) in dark steamed wood indicates that the thermodegradation products (formed during steaming in an autoclave) are less stable to UV radiation than native lignin and that their gradual decomposition or lightening occurs under the action of light.

The results show that although steaming successfully homogenizes the color between sapwood and false heartwood, their subsequent photochemical stability remains affected by the intensity of the thermal treatment. While at 105 °C, the processes of formation of new chromophores dominate (darkening), at 120 °C, the degradation processes of existing dyes prevail (lightening).

**Figure 2:**
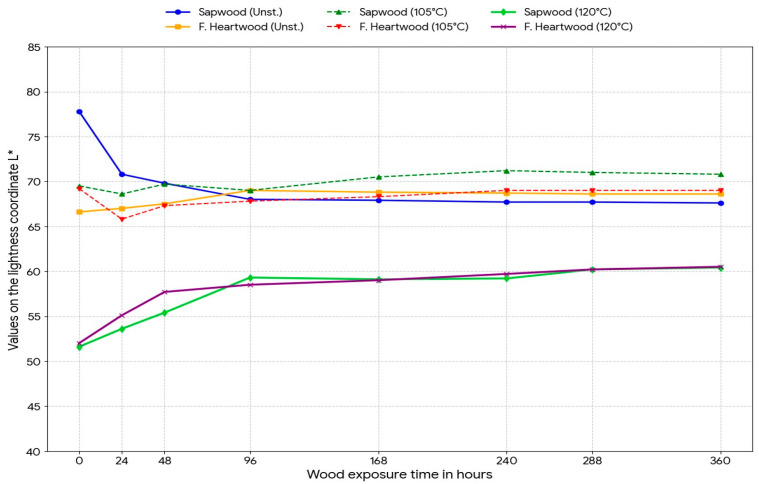
Kinetics of changes in the lightness coordinate L* of beech sapwood and false heartwood depending on the time of UV exposure.

**Figure 3:**
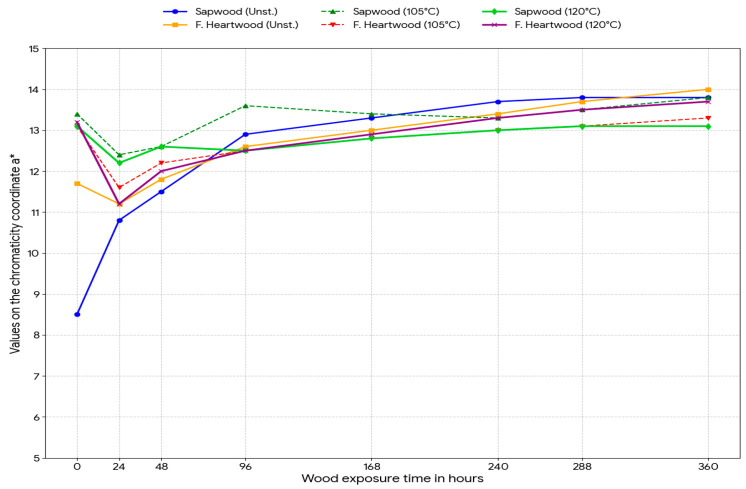
Effect of UV irradiation duration on changes in the chromatic coordinate a* in unsteamed and steamed samples.

**Figure 4:**
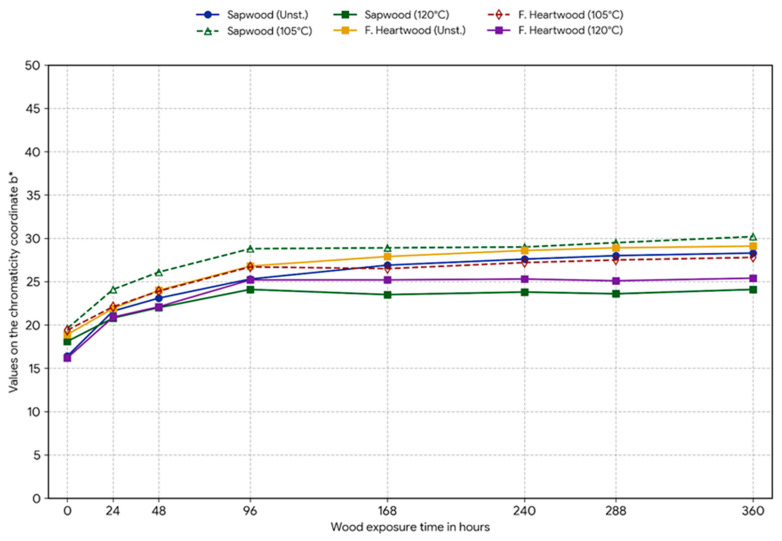
The course of changes in the chromatic coordinate b*, indicating the degree of photochemical yellowing of the sample surface during 360 h of exposure.

**Figure 5:**
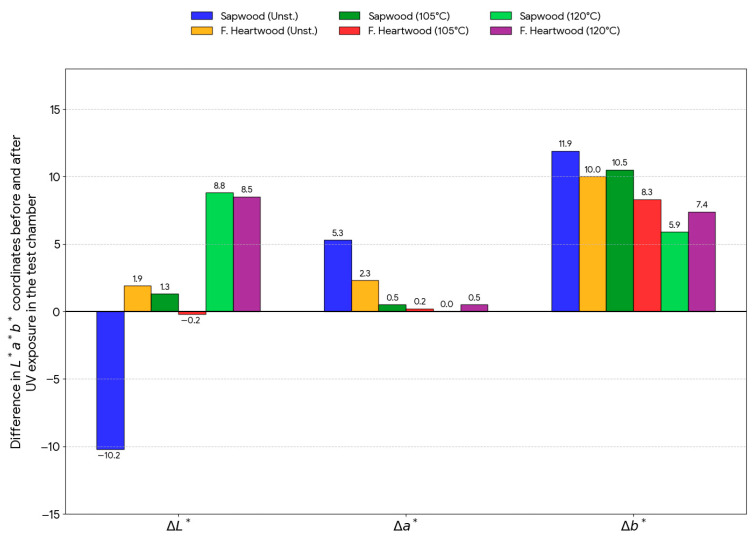
Total differences in color coordinates ΔL*, Δa*, and Δb* after the end of simulated aging for individual beech wood variants.

Experimental data obtained by measuring color coordinates in the CIE Lab* space were subjected to statistical analysis of variance (ANOVA) to determine the influence of two factors: steaming mode and length of UV exposure, and were graphically drawn in Figure 2, Figure 3, Figure 4 and Figure 5.

The analysis confirmed that before irradiation, there was a significant difference (*p* < 0.001) in lightness between unsteamed white (77.8 ± 1.9) and false core (66.6 ± 1.7).

Unsteamed samples: UV radiation caused a sharp and statistically significant decrease in lightness L* in the first 96 h of exposure (from 77.8 to approximately 68.0). The total decrease in lightness in white was 10.2 units.

Steamed samples (Mode II): The statistical model showed the opposite trend, where, after the initial phase, a lightening of the surface occurred. The lightness of samples steamed at 120 °C increased by 8.8 (sapwood) and 8.5 (false heartwood), indicating suppression of darkening due to the previous heat treatment.

Coordinate a*: An initial decrease was recorded for unsteamed sapwood, followed by an increase of 5.3 units, which is visually perceptible as browning. For steamed wood (Mode II), this change was statistically insignificant (0.0 to 0.5), indicating a high stability of the red shade after steaming.

Coordinate b*: All samples showed a statistically significant increase in yellowness. The highest photochemical yellowing rate was measured for unsteamed sapwood (Δb* = 11.9) and for steamed sapwood at 105 °C (Δb* = 10.5).

Visual and physical changes in the color of the wood surface are a direct consequence of photochemical reactions initiated by UV radiation in the wavelength range λ = 200–400 nm. The main recipient of this radiation is lignin, which absorbs up to 80–85% of the incident energy, leading to its degradation and the formation of new chromophoric groups (carbonyl, carboxyl groups, and free radicals) [15,41].

The significant darkening of unsteamed wood is in accordance with the works of [20,41], who attribute this phenomenon to the formation of new chromophoric structures, such as quinones and carbonyl groups.

An interesting finding is the behavior of samples steamed at a higher temperature (120 °C). These samples lighten after exposure to UV radiation. This phenomenon may be explained by the fact that higher steaming temperatures are associated with a more pronounced disruption of the lignin–carbohydrate complex, thereby making lignin more accessible to subsequent photochemical processes. Consequently, the formation of chromophoric structures increases, which initially manifests as darker coloration of the wood [42]. However, these newly formed chromophores are less resistant to UV radiation than native lignin and are subject to lightening or further photochemical decomposition upon exposure to light, which leads to an increase in the lightness L*.

**Figure 6:**
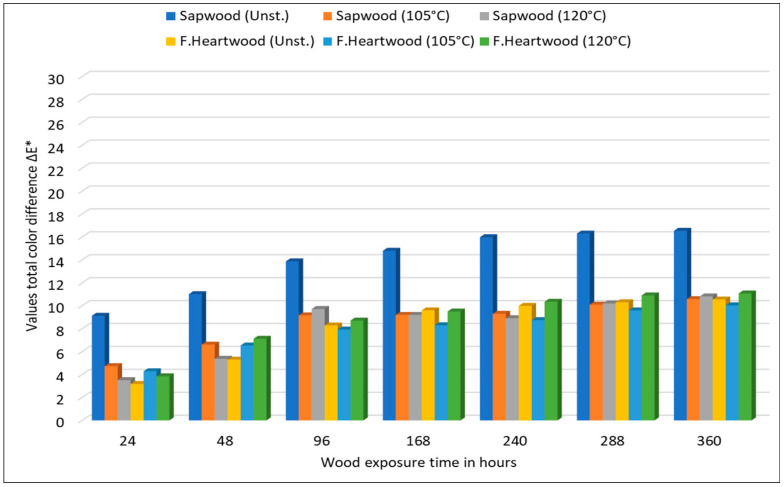
Influence of UV radiation on the size of the total color difference ΔE* of unsteamed and steamed wood.

Analysis of the ΔE* graph (Figure 6) for unsteamed and steamed beech samples reveals fundamental differences in the kinetics and intensity of their photodegradation. The ΔE* graph shows that the most drastic changes occur in the initial phase of exposure (first 48–96 h). After just 24 h of irradiation in the Q-SUN chamber, the ΔE* values for all variants exceed the threshold of 3.0, which is classified as a “very noticeable change” according to the *National Bureau of Standards (NBS) criteria* [43].

For unsteamed sapwood, the ΔE* reaches values in the range of 12 to 15 units after 360 h, which represents a “completely different color” compared to the original state. This steep increase is in line with the work of [44], who state that native lignin on the surface of hardwoods undergoes immediate photooxidation to form dark chromophoric products.

The graphs clearly differentiate the surface resistance according to the steaming temperature:

Steaming at 105 °C (Mode I): These samples show slightly lower final ΔE* values than unsteamed wood, but the trend is still increasing, dominated mainly by the increase in yellowness (Δb*).

Steaming at 120 °C (Mode II): Although the initial ΔE* value is high, the curve tends to stabilize early. Unlike unsteamed wood, where ΔE* is driven by darkening, in the 120 °C mode, the resulting difference is the result of lightening. This fact is important from a design perspective: while unsteamed beech turns brown-gray over time, steamed beech at higher temperatures retains its brightness and only changes its color undertone.

From an industrial perspective, the most significant finding is the convergence of the ΔE* curves for sapwood and false heartwood for steamed samples. While in the untreated state, the difference in their photochemical response is low (sapwood visually degrades faster), the steaming process increases these differences. Statistically insignificant differences between ΔE* of sapwood and false heartwood at 120 °C confirm that heat treatment not only unifies the initial shade, but also ensures uniform visual aging of the wood surface.

**Figure 7:**
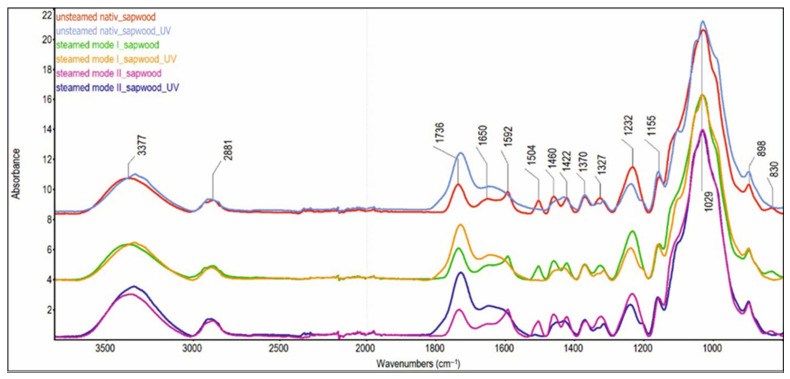
ATR-FTIR spectra of unsteamed and steamed sapwood of beech before and after UV irradiation.

**Figure 8:**
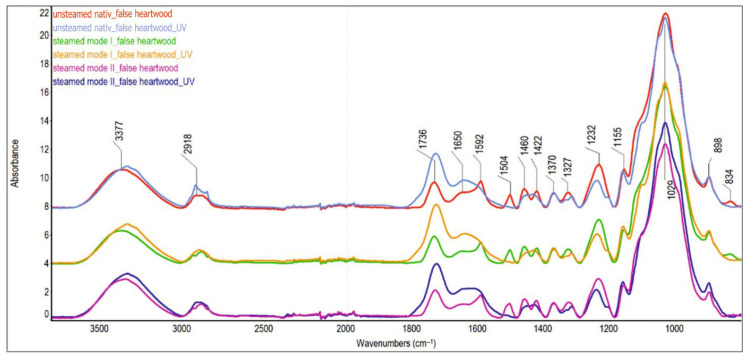
ATR-FTIR spectra of unsteamed and steamed false heartwood of beech before and after UV irradiation.

In order to identify chemical changes in beech sapwood and false heartwood, ATR-FTIR analysis was performed (Figure 7 and Figure 8).

In unsteamed wood, the proportion of aromatic structures is higher in the false heartwood than in the sapwood. This is indicated by the higher relative intensity values of the H_1592_/H_1370_ and H_1504_/H_1370_ cm^−1^ bands in the false heartwood spectrum (1.89 and 0.93, respectively) compared with the sapwood spectrum (1.44 and 0.85, respectively). Lignin contributes mainly to the intensity of these absorption bands; however, to a lesser extent, aromatic nuclei from some groups of extractives may also contribute.

As a result of steaming, the absorption band with a maximum at 1504 cm^−1^ shifts to a higher wavenumber. A detailed analysis of the spectra revealed that during the steaming process, especially in mode II, a new absorption band at 1518 cm^−1^ is formed, which overlaps with the original band at 1504 cm^−1^. This suggests that pseudo-lignin is formed by condensation of polysaccharide degradation products. Similar aromatic aggregates formed during thermal or hydrothermal processing have been reported by other authors [45,46,47,48].

After irradiation, a rapid decrease or complete disappearance of the absorption bands at 1504 cm^−1^, 1461 cm^−1^, 1423 cm^−1^, 1326 cm^−1^, and 1235 cm^−1^ was observed. This is mainly associated with the degradation of lignin. This is in agreement with [49], who found a statistically significant relationship between lignin decay indicated by the I_1507_/I_1375_ peak ratio and the chromatic coordinates L* and a* under the artificial photo-irradiation of normal and red heart beech wood.

The change in the shape of the absorption band and its intensity is also evident in the wavenumber range between 1350 and 1290 cm^−1^. While in the spectra of wood before irradiation, a single absorption band with a maximum at 1327 cm^−1^ is present in this region, in the spectra of wood after irradiation, a doublet with maxima at 1333 and 1316 cm^−1^ is observable, with the band at 1316 cm^−1^ being more intense. These changes indicate the degradation of the S-ring plus the condensed G-ring in the lignin macromolecule, since the band at 1333 cm^−1^ belongs to the S-ring plus the condensed G-ring in the lignin macromolecule and the band at 1316 cm^−1^ to the CH_2_ wagging in cellulose.

Other significant changes in the FTIR spectrum induced by UV radiation relate to the absorption bands of carbonyl groups (Table 3). The absorption band of unconjugated C=O groups at 1736 cm^−1^, and the band of conjugated C=O and C=C, and aromatic ketones at 1650 cm^−1^ increase markedly in all samples under the influence of UV radiation. Changes in the intensity of the absorption band at 1736 cm^−1^ are influenced by several processes, which often have opposing effects on this band. On the one hand, the degradation of hemicelluloses, which preferentially occurs during steaming, leads to a decrease in their intensity. On the other hand, oxidation reactions—occurring mainly due to UV irradiation—lead to the formation of new carbonyl and carboxyl groups, which results in an increased intensity of the absorption bands at 1736 cm^−1^ and 1650 cm^−1^.

It is known that lignin is the most UV-sensitive component among all wood constituents [22,30,34]. Degradation of lignin’s aromatic bonds results in the generation of free radicals. These radicals subsequently react with oxygen, yielding carbonyl groups [50]. However, the decrease in lignin on the wood surface also disrupts the lignin–carbohydrate complex and makes the cellulose structure accessible to UV radiation, thereby improving the conditions for cellulose oxidation [45,49].

The observed relationships between changes in characteristic lignin absorption bands (evaluated via FTIR peak ratios) and chromaticity coordinates suggest that the total color difference (ΔE*) serves as a reliable indicator of photochemical degradation within the lignin–carbohydrate complex on beech wood surfaces. Our analysis demonstrates a strong correlation between ΔE* kinetics and the intensity of surface photochemical transformations. Specifically, high ΔE* values across all sample types correspond directly to the decrease in absorbance within the aromatic skeleton regions of the FTIR spectra.

## 4. Conclusions

The research confirmed that the steaming of beech wood with false heartwood significantly affects its visual and chemical response to UV radiation. While unsteamed beech wood undergoes intense yellowing and darkening due to photooxidation of native lignin, wood steamed at 120 °C shows a lightening, which is caused by the decomposition of less stable chromophores formed during the steaming process. Steaming at this higher temperature not only successfully unifies the initial color shade between sapwood and false heartwood, but also ensures their statistically identical visual aging over time. In addition, high stability of the red shade was recorded for wood steamed at 120 °C, while changes in the a* coordinate due to UV radiation were statistically insignificant. From the point of view of industrial use in interiors, it can be stated that the most suitable technology for achieving a color-stable and visually uniform surface of beech material with false heartwood is steaming with saturated water steam at a temperature of 120 °C.

## Figures and Tables

**Table 1:** Parameters of pressure steaming for beech wood color unification.

Steaming Modes	Conditions for Steaming Beech Wood		
	Temperature [°C]	Pressure [MPa]	Time [h]
Mode I	105	0.122	18
Mode II	120	0.199	9

**Table 2:** Color space CIE L*a*b* coordinate values of unsteamed and steamed beech wood before irradiation.

	Visual Assessment of Wood Color	Number of Measurements	Color Coordinates in the Color Space CIE L*a*b*		
			L*	a*	b*
Unsteamed sapwood	light white-gray-yellow color	60	77.8 ± 1.9	8.5 ± 1.0	16.3 ± 1.6
Unsteamed false heartwood	pale pink-brown color	60	66.6 ± 1.7	11.7 ± 1.1	18.8 ± 1.4
Steamed mode I_sapwood	red-brown color	60	69.5 ± 1.2	13.4 ± 0.8	19.6 ± 1.0
Steamed mode I_false heartwood		60	69.2 ± 1.5	13.1 ± 0.8	19.4 ± 1.2
Steamed mode II_sapwood	rich brown-red color	60	51.6 ± 1.2	13.1 ± 0.9	18.1 ± 1.0
Steamed mode II_false heartwood		60	52.0 ± 1.4	13.2 ± 0.7	18.2 ± 0.9

**Table 3:** Relative intensities of absorption bands of carbonyl groups in infrared spectra of unsteamed and steamed beech wood.

	H_1736_/H_1370_	H_1650_/H_1370_
Unsteamed_sapwood	1.87	0.94
Unsteamed_sapwood_UV	3.82	1.59
Unsteamed_false heartwood	1.80	1.09
Unsteamed_false heartwood_UV	3.82	1.94
Steamed mode I_sapwood	2.08	0.95
Steamed mode I_sapwood_UV	3.74	1.71
Steamed mode I_false heartwood	1.90	0.86
Steamed mode I_false heartwood_UV	4.05	2.00
Steamed mode II_sapwood	1.79	0.83
Steamed mode II_sapwood_UV	4.17	1.98
Steamed mode II_false heartwood	1.88	0.93
Steamed mode II_false heartwood_UV	3.74	1.99

## Data Availability

The original contributions presented in this study are included in the article. Further inquiries can be directed to the corresponding author.
